# The Composite Face Effect Between Young and Older Chinese Adults Remains Stable

**DOI:** 10.3389/fpsyg.2021.743056

**Published:** 2021-12-09

**Authors:** Lina Zhang, Qi Yang, Werner Sommer, Changming Chen, Guiting Guo, Xiaohua Cao

**Affiliations:** ^1^Department of Psychology, Zhejiang Normal University, Jinhua, China; ^2^School of Humanities, Tongji University, Shanghai, China; ^3^Department of Psychology, Humboldt-Universität zu Berlin, Berlin, Germany; ^4^School of Education, Chongqing Normal University, Chongqing, China; ^5^Division of Student Affairs, Sanming University, Sanming, China; ^6^Key Laboratory of Intelligent Education Technology and Application of Zhejiang Province, Zhejiang Normal University, Jinhua, China

**Keywords:** older, holistic processing, Chinese, development, composite face effect

## Abstract

Holistic face perception is often considered to be a cornerstone of face processing. However, the development of the ability to holistically perceive faces in East Asian individuals is unclear. Therefore, we measured and compared holistic face processing in groups of Chinese children, young adults, and older adults by employing the complete composite face paradigm. The results demonstrate a similar magnitude of the composite effect in all three groups although face recognition performance in the task was better in young adults than in the two other groups. These findings suggest that holistic face perception in Eastern individuals is stable from late childhood to at least age 60, whereas face memory may be subject to later development and earlier decline.

## Introduction

Humans are visual experts in face perception (Richler and Gauthier, 2014). A key characteristic of face perception is its holistic nature (Gauthier et al., 2003; Richler and Gauthier, 2014), that is, processing the face as a whole rather than in a piecemeal and feature-based fashion. Holistic face perception has been widely explored through the composite face paradigm (Richler and Gauthier, 2014), which combines the top and bottom halves of different faces to create a new “composite” face. Participants are asked to evaluate the top (or bottom) half of a composite face (target) while ignoring the bottom (or top) half (non-target) and judge whether the target half of the study face is the same or different relative to the test face. In congruent trials, the response to the target part (top or bottom) matches the same/different status of the non-target part (i.e., both parts are the same or both parts are different). In incongruent trials, the response to the target part conflicts with the same/different status of the non-target part (i.e., one part is the same and the other part is different). Holistic processing is often inferred from the interaction between congruency and alignment (e.g., Richler et al., 2011): Performance is better in congruent than incongruent trials, and the magnitude of this congruency effect is reduced when parts are misaligned. This process has been termed the composite face effect, which could be interpreted as a failure of selective attention to face parts (Richler et al., 2008) or as a combination of various facial features into a gestalt (Rossion, 2013).

Studies have investigated holistic face processing with the composite face paradigm in both children and adults (Gauthier et al., 2003; Schwarzer et al., 2010; Meinhardt-Injac et al., 2017). Developmental studies have demonstrated that young children already possess holistic face processing ability (De Heering et al., 2007; Ventura et al., 2018) and reach an adult-like level by age 11 in both Caucasians (Durand et al., 2007; Petrakova et al., 2018) and East Asian individuals (Sun et al., 2020). Importantly, recent studies explored the characteristics of holistic face processing across the lifespan (Cheng et al., 2016; Meinhardt-Injac et al., 2017) and demonstrated that the holistic face processing ability is stable from 11 years of age to adulthood (De Heering et al., 2007; Ventura et al., 2018; Sun et al., 2020). However, findings about the stability of holistic face processing in the age range of 60–85 years have been inconsistent (Boutet and Faubert, 2006; Konar et al., 2013; Wiese et al., 2013; Meinhardt-Injac et al., 2014, 2017; Cheng et al., 2016; Boutet and Meinhardt-Injac, 2018). For example, many studies revealed that the elderly of 60–80 years show a similar face composite effect as young adults (Boutet and Faubert, 2006; Konar et al., 2013; Meinhardt-Injac et al., 2014; Cheng et al., 2016). In contrast, other studies showed that, relative to young adults, the magnitude of holistic face processing effects for people aged 60–85 years declines significantly (Boutet and Faubert, 2006, Exp.3; Wiese et al., 2013; Meinhardt-Injac et al., 2017). There are many possible reasons for these inconsistencies. First, from 60 to 85 years, many abilities, such as face recognition and attention span decline (Crook and Larrabee, 1992; Lamont et al., 2005; Boutet and Faubert, 2006; Hildebrandt et al., 2010; Germine et al., 2011). For example, the attention span of the elderly over age 75 was found to be significantly worse than that of people below age 75 (Greenwood and Parasuraman, 1994; Reuter et al., 2016). Importantly, face recognition abilities decrease between ages 60 to 85 years (Benton et al., 1981; Crook and Larrabee, 1992; Lamont et al., 2005; Hildebrandt et al., 2010; Cheng et al., 2016, Study 2). Therefore, the exploration of holistic face processing ability between ages 60–85 years may be confounded with age-related changes in face recognition and attention-related abilities. Importantly, there is evidence that the face recognition and attention-related abilities in the young elderly between ages 56–65 years are similar to those in young adults (Crook and Larrabee, 1992; Greenwood and Parasuraman, 1994). Therefore, it is of interest to focus on a relatively narrow range of young older adults (e.g., 56–65 years) to determine whether holistic face processing remains stable compared with young adults.

Second, the details of the composite face paradigm were different in the above studies in terms of the presentation duration of the study face, the partial or complete composite paradigm, and so on. Specifically, in some studies, the presentation duration of the study face was only 200 ms (Konar et al., 2013), while in others, it was up to 600–800 ms (Wiese et al., 2013; Meinhardt-Injac et al., 2014). Most of the studies adopted a partial composite face paradigm (Konar et al., 2013; Wiese et al., 2013; Meinhardt-Injac et al., 2017); few used a complete composite face paradigm (Cheng et al., 2016), which was found to be insusceptible to response bias unrelated to holistic processing (Richler et al., 2011). The procedural differences of these paradigms make it difficult to account for the inconsistent results of previous studies.

Additionally, most of the participants in the studies mentioned above were Westerners (Boutet and Faubert, 2006; Konar et al., 2013; Wiese et al., 2013; Meinhardt-Injac et al., 2014, 2017; Boutet and Meinhardt-Injac, 2018; except for Cheng et al., 2016, East Asian adults). Research has indicated that Asian adults outperform their Western counterparts in holistic face processing (Lewis et al., 2008; Miyamoto et al., 2011). For instance, Japanese participants performed better in holistic processing versus feature identification in comparison with American participants when matching prototypical faces (Miyamoto et al., 2011). Moreover, Asians show strong holistic processing of faces for both own-race and Caucasian other-race faces, whereas Caucasians demonstrate better holistic processing for Caucasian rather than other-race faces (Michel et al., 2006; Crookes et al., 2013). Hence, the evidence that holistic processing in Asians is better than in Western individuals appears to be consistent. However, little is known about the stability of holistic face processing between young adults and older adults in Asians.

To summarize, our knowledge about the development of holistic face processing across the lifespan is incomplete (Cheng et al., 2016; Meinhardt-Injac et al., 2017). Studies have shown that holistic face processing ability in East Asian individuals is similar from 11 years of age to adulthood (Sun et al., 2020). However, it is unknown whether the holistic face processing ability is stable from late childhood to older adulthood in East Asian individuals. Therefore, in this study, we recruited three age groups of Chinese people (children aged 11–13 years, young adults aged 23–26 years, and older adults aged 56–65 years) and employed the complete composite paradigm using both a 200 ms and 600 ms stimulus presentation duration. Based on previous evidence that face recognition and attention-related abilities of older adults aged 56–65 years and young adults are similar (Crook and Larrabee, 1992; Greenwood and Parasuraman, 1994), any age-effects in holistic processing should be independent of age-related declines in attention or face memory. Likewise, it was of interest to assess differences in holistic processing between children and adults, which might be expected on the basis of stronger holistic processing in Asian participants. Therefore, we tested whether holistic face processing ability is stable from late childhood to about age 60 in Eastern individuals.

## Materials and Methods

### Participants

Twenty children (11 female; mean age = 12.10 years, age range 11–13), twenty-one young adults (9 female; mean age = 24.19 years, age range 23–26), and twenty-three older adults (14 female; mean age = 61.3 years, age range 56–65) were recruited. One young adult was excluded from further analysis due to their accuracy being less than chance level (0.5), and one child was excluded because his average response time was outside the three standard deviations of the same group. The final sample consisted of 62 subjects, including 19 children, 20 young adults, and 23 older adults. We used G-Power 3.1 (Faul et al., 2009) to calculate the required sample sizes. For the measurement of the composite effect using the complete design, a meta-analysis found an average effect size of $\eta_{p}^{2}$ = 0.32 (Richler and Gauthier, 2014). A power analysis indicated that a sample size of 15 in one group would be required to detect this effect size at the 0.05 alpha level with 80% power. All participants were right-handed native Chinese, with normal or corrected-to-normal vision. Written or verbal consent was obtained from all participants, and the ethical committee of Zhejiang Normal University approved the study.

### Stimuli

**Figure 1:**
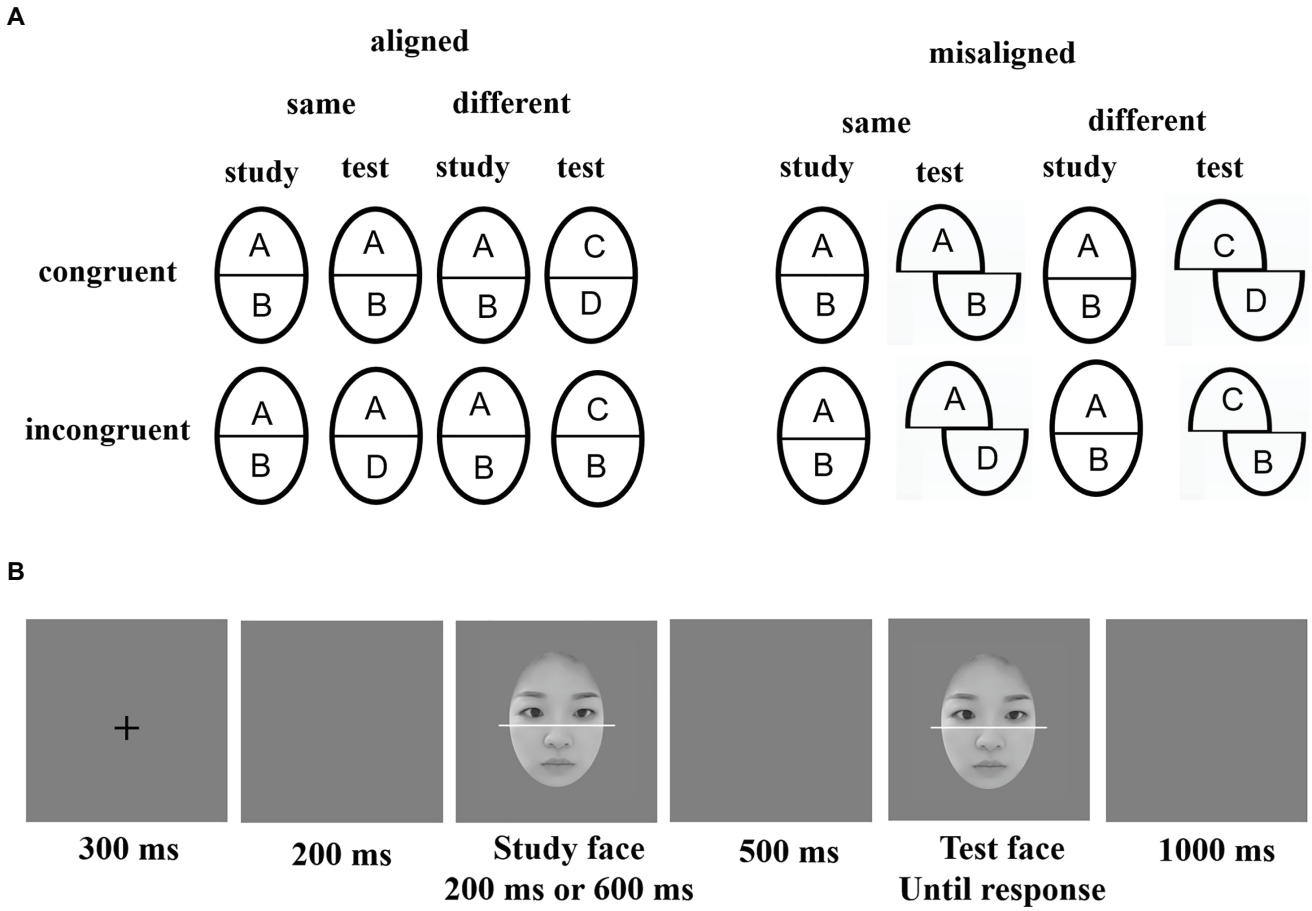
**(A)** Schematic diagram of the composite face task in a complete design. **(B)** Example for the aligned-congruent condition for one trial. Participants need to pay attention to the top half of the study face, while ignoring the bottom half, and indicate whether the top part of the test face is the same as the study face. The study faces are always presented in aligned form, and the test faces may be presented in aligned or misaligned form.

The original materials used were 20 pictures of faces from Chinese adults aged 18–23 years with neutral expressions (10 male and 10 female faces; see Figure 1). Using Adobe Photoshop, the images were edited by removing all external facial features, such as hair and ears, and the isolated faces were placed against a neutral gray background. Each picture had a similar level of brightness and had dimensions of 185 × 230 pixels, corresponding to a visual angle of 5.89° × 6.66°. A 3-pixel wide white line was used to split the faces into equal top and bottom halves, which were then randomly assigned top and bottom halves from different faces to create 20 aligned and 20 misaligned composite faces (i.e., two images of the same gender were paired to form a new face). For the aligned faces, the top and bottom halves were aligned to form a new face; and for the misaligned faces, the lower halves of the faces were moved to the right by 60 pixels. Regardless of whether the face was aligned, the top half was always presented in the center of the screen.

### Procedure

During the experiment, the subjects sat 60 cm from the computer screen. In order to familiarize the participants with the experimental process, 16 trials of practice experiments were carried out before the formal experiment. We used E-prime 1.0 to present stimuli on a 14-inch Lenovo G470 laptop for experiments.

Each trial started with a fixation cross displayed at the center of the screen (300 ms), followed by a blank screen (200 ms). Next, a study composite face was displayed (200 ms or 600 ms), followed by a blank screen (500 ms). After that, a test composite face was presented until the participant responded. The inter-trial interval was 1,000 ms (see Figure 1). The participants were told to ignore the bottom halves of the faces and focus only on the top halves to judge whether the top-half pairs of the study face and test face were the “same or different.” Half of the participants were told to press “A” for “same” or “L” for “different;” for the other half, the key-pressing requirement was reversed.

This experiment comprised 320 trials (i.e., eight blocks of 40 trials). There were 160 trials with a 200 ms and 600 ms stimulus presentation duration, respectively. Each presentation had four conditions [2(alignment: aligned vs. misaligned) × 2(congruency: congruent vs. incongruent)] in each block, which included 10 trials per condition. In aligned trials, both study faces and test faces were aligned; in misaligned trials, study faces were aligned while the test faces were misaligned. In congruent trials, the response to the target part matched the same/different status of the irrelevant part (i.e., both parts were the same or both parts were different). In incongruent trials, when the relevant part was the same, the irrelevant part was different (and *vice-versa*).

### Data Analysis

The data were analyzed using a 3 (Subject Group: children vs. young adults vs. older adults) × 2 (Alignment: aligned vs. misaligned) × 2 (Congruency: congruent vs. incongruent) × 2 (Stimulus Presentation Duration: 200 ms vs. 600 ms) repeated ANOVA with stimulus presentation duration, alignment, and congruency as the within-subject factors and subject group as the between-subject factor. We deleted 1.8% of trials in which the response time was less or longer than three standard deviations (SD) above the mean based on each participant.

The dependent measures were mean sensitivity (A′) and response time. A′ represents response sensitivity for each condition based on the signal detection theory. Sensitivity is widely used and relatively unaffected by response bias when the assumptions of normality and equal variances are violated (Verde et al., 2006). Therefore, it is appropriate for evaluating the pure composite face effect. A′ was computed using the following formula (Stanislaw and Todorov, 1999):


$$A'=0.5+sign\left(H-F\right)\;\frac{{\left(H-F\right)}^{2}+|H-F|}{4\max\left(H,F\right)-4HF}$$

In this formula, H represents the hit rate, and F refers to the false alarm rate. The response time was calculated as the correct response time between the onset of the test stimuli and the participant’s response. In the present study, holistic processing is inferred from the interaction between congruency and alignment.

## Results

**Figure 2:**
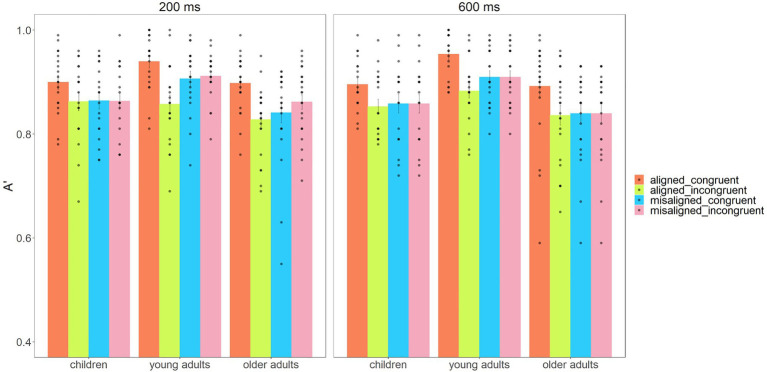
Mean sensitivities (A') and standard errors for alignment, congruency, and stimulus presentation duration as a function of children, young adults, and older adults.

**Figure 3:**
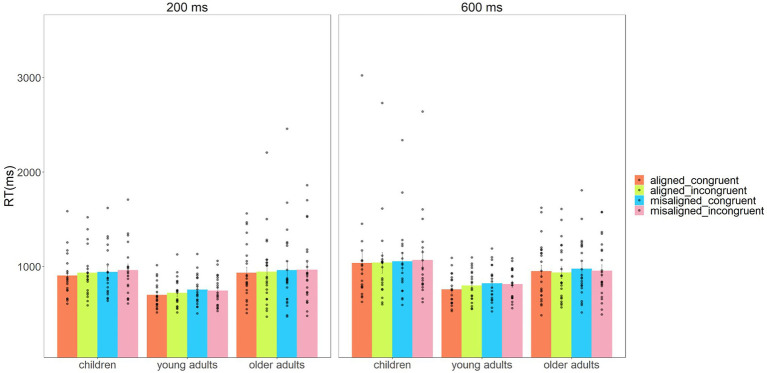
Mean response time (RT) and standard errors for alignment, congruency, and stimulus presentation duration as a function of children, young adults, and older adults.

The descriptive results of mean sensitivities (A′) and response times are shown in Figures 2, 3.

### Analysis of A'

The analysis of the sensitivities (A′) yielded a significant main effect for the Subject Group, *F*(2,59) = 6.610, *p* = 0.003, $\eta_{p}^{2}$ = 0.183, BF_10_ = 13.143. A *post hoc t* test revealed a higher sensitivity for young adults (*M* = 0.91) compared to both children (*M* = 0.87, *t*_(37)_ = 2.814, *p* = 0.008, Cohen’s *d* = 0.925) and older adults (*M* = 0.85, *t*_(41)_ = 3.510, *p* = 0.001, Cohen’s *d* = 1.096; multiple comparisons were Bonferroni-adjusted with *p* value = 0.017 due to three-pair sample comparisons). The results revealed the main effect for Congruency, *F*(1,59) = 25.626, *p* < 0.001, $\eta_{p}^{2}$ = 0.303, BF_10_ = 0.727. We neither found a main effect for Alignment, *F*(1,59) = 3.786, *p* = 0.056, $\eta_{p}^{2}$ = 0.060, BF_10_ = 0.618, nor for Stimulus Presentation Duration, *F*(1,59) = 0.004, *p* = 0.950, $\eta_{p}^{2}$<0.001, BF_10_ = 0.098.

**Figure 4:**
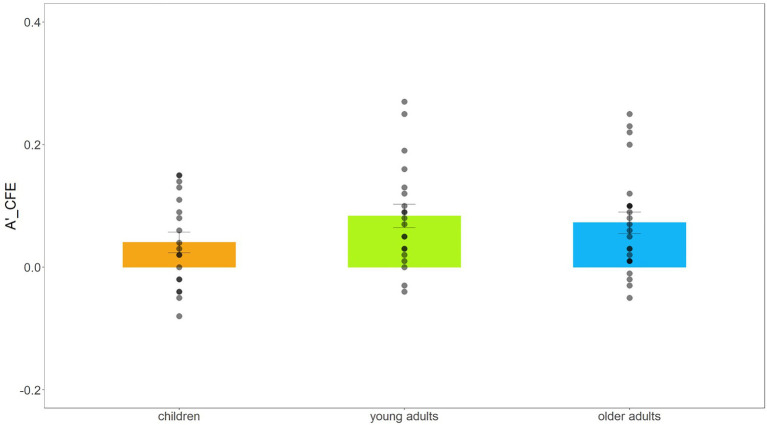
Bar plot with data of CFEA' (A'_aligned congruent_ – A'_aligned incongruent_) – (A'_misaligned congruent_– A'_misaligned incongruent_) and standard errors of children, young adults, and older adults. The bar plot in the figure shows the mean size of CFEA' of each group, and the block dots the are the data distribution across participants.

It is important to note that there was a significant two-way interaction of Alignment × Congruency, *F*(1,59) = 39.413, *p* < 0.001, $\eta_{p}^{2}$ = 0.400, and that the *post hoc t* test revealed higher sensitivity in congruent compared to incongruent trials when faces were aligned, *t*_(61)_ = 6.677, *p* < 0.001, Cohen’s *d* = 1.710, and similar sensitivity in both consistent and inconsistent trials in the misaligned condition, *t*_(61)_ = 1.176, *p* = 0.244, Cohen’s *d* = 0.301. This result showed that the face composite effect was observed in all three groups (also see Supplementary Material). To visualize the composite face effect across age groups, we used the CFE*A*′ index, defined as [(A′_aligned congruent_ – A′_aligned incongruent_) – (A′_misaligned congruent_– A′_misaligned incongruent_)]. The CFE*A*′ index reflects the dependency of the interference due to irrelevant face parts on the intactness of the face configuration (Bukach et al., 2010; Richler et al., 2011). The visualization data of CFE*A′* are shown in Figure 4.

In order to provide an index of the strength of evidence that the participants had strong holistic face processing ability, we turned to Bayesian analysis. We used Bayesian repeated measures ANOVA on the sensitivity (A′) to assess the likelihood of the null hypothesis H_0_ (the interaction is absent) over H_1_ (the interaction is present) for the interaction of Alignment × Congruency. The Bayes factor (BF_10_) is the ratio of the amount of evidence for H_1_ above H_0_. The Bayesian analysis provided strong evidence for the alternative hypothesis (H_1_), BF_10_ = 1.923*10^7^. These results, combined with those from the traditional ANOVA, establish a reliable pattern of results, indicating that the interaction between Alignment and Congruency was present. The analysis was performed with JASP.^1^

Other two-way interactions were not significant (Subject Group × Alignment, *F*(2,59) = 1.508 *p* = 0.230, $\eta_{p}^{2}$ = 0.049, BF_10_ = 0.194; Subject Group × Congruency, *F*(2,59) = 0.734, *p* = 0.484, $\eta_{p}^{2}$ = 0.024, BF_10_ = 0.071; Subject Group × Stimulus Presentation Duration, *F*(2,59) = 0.786, *p* = 0.460, $\eta_{p}^{2}$ = 0.026, BF_10_ = 0.108; Alignment × Stimulus Presentation Duration, *F*(1,59) = 0.920, *p* = 0.342, $\eta_{p}^{2}$ = 0.015, BF_10_ = 0.150; Congruency × Stimulus Presentation Duration, *F*(1,59) = 0.040, *p* = 0.843, $\eta_{p}^{2}$ = 0.001, BF_10_ = 0.136).

Additionally, there was no three-way interaction among Subject Group × Alignment × Congruency, *F*(2,59) = 1.407, *p* = 0.253, $\eta_{p}^{2}$ = 0.046, which means that the magnitude of the holistic face processing effect was indistinguishable between the three groups. Due to the null effect of the three-way interaction, we turned to the Bayesian analysis to provide an index of the strength of evidence for the absence of differences in holistic face processing of children, young adults, and older adults. The Bayesian repeated measures ANOVA was applied to estimate the strength of evidence for the presence of differences in holistic face processing between Subject Groups (H_1_) as compared to evidence for the absence of such differences (H_0_). The results showed medium evidence for the null hypothesis (H_0_), BF_10_ = 0.325. These results, along with those from the traditional ANOVA, establish a reliable pattern that there are no differences in the magnitude of holistic face effect between the three subject groups.

There were no other three-way interactions (Subject Group × Alignment × Stimulus Presentation Duration, *F*(2,59)= 0.495, *p* = 0.612, $\eta_{p}^{2}$ = 0.017, BF_10_ = 0.143; Subject Group × Congruency × Stimulus Presentation Duration, *F*(2,59) = 0.119, *p* = 0.888, $\eta_{p}^{2}$ = 0.004, BF_10_ = 0.097; Alignment × Congruency × Stimulus Presentation Duration, *F*(1,59) = 0.442, *p* = 0.509, $\eta_{p}^{2}$ = 0.007, BF_10_ = 0.194). Finally, there was also no four-way interaction, *F*(2,59) = 0.807, *p* = 0.451, $\eta_{p}^{2}$ = 0.027, BF_10_ = 0.160.

### Analysis of Response Time

**Figure 5:**
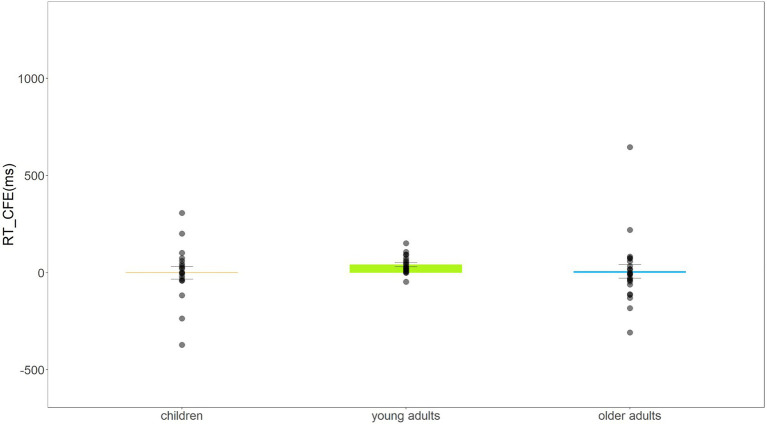
Bar plot with data of CFERT (RT_aligned congruent_ – RT_aligned incongruent_) – (RT_misaligned congruent_ – RT_misaligned incongruent_) and standard errors of children, young adults, and older adults. The bar plot in the figure showed the mean size of CFERT of each group, and the black dots were the original data distribution of each participant.

The analysis of the response time revealed a significant main effect of the Subject Group, *F*(2,59) = 3.670, *p* = 0.031, $\eta_{p}^{2}$ = 0.111, BF_10_ = 2.083. A *post hoc t* test corrected by the Bonferroni method revealed a marginally significant quicker response for young adults (762 ms) compared to both children (*M* = 992 ms, *t*_(37)_ = 2.675, *p* = 0.011, Cohen’s d = 0.880) and older adults (*M* = 952 ms, *t*_(41)_ = 2.462, *p* = 0.018, Cohen’s *d* = 0.769). The results revealed that the effect for Alignment, *F*(1,59) = 13.205, *p* = 0.001, $\eta_{p}^{2}$ = 0.183, BF_10_ = 1.800, was a faster response time for the aligned condition (926 ms) than for the misaligned condition (953 ms). The main effect of Congruency was not significant, *F*(1,59) = 0.792, *p* = 0.377, $\eta_{p}^{2}$ = 0.013; BF_10_ = 0.091. In the case of the Stimulus Presentation Duration, *F*(1,59) = 5.459, *p* = 0.023, $\eta_{p}^{2}$ = 0.085, BF_10_ = 4038.772, the response time was slower for the 600 ms presentation duration (*M* = 932 ms) compared to the 200 ms presentation duration (*M* = 871 ms). There were no other significant interactions (Subject Group × Alignment, *F*(2,59) = 0.292, *p* = 0.748, $\eta_{p}^{2}$ = 0.010, BF_10_ = 0.760; Subject Group × Congruency, *F*(2,59) = 0.658, *p* = 0.522, $\eta_{p}^{2}$ = 0.022, BF_10_ = 0.043; Subject Group × Stimulus Presentation Duration, *F*(2,59) = 1.495, *p* = 0.233, $\eta_{p}^{2}$ = 0.048, BF_10_ = 15.000; Alignment × Congruency, *F*(1,59) = 0.849, *p* = 0.360, $\eta_{p}^{2}$ = 0.014, BF_10_ = 0.200; Alignment × Stimulus Presentation Duration, *F*(1,59) = 0.074, *p* = 0.786, $\eta_{p}^{2}$ = 0.001, BF_10_ = 0.136; Congruency × Stimulus Presentation Duration, *F*(1,59) = 0.482, *p* = 0.490, $\eta_{p}^{2}$ = 0.008, BF_10_ = 0.143; Subject Group × Alignment × Congruency, *F*(2,59) = 0.519, *p* = 0.598, $\eta_{p}^{2}$ = 0.017, BF_10_ = 0.118; Subject Group × Alignment × Stimulus Presentation Duration, *F*(2,59) = 0.041, *p* = 0.960, $\eta_{p}^{2}$ = 0.001, BF_10_ = 0.069; Subject Group × Congruency × Stimulus Presentation Duration, *F*(2,59) = 0.519, *p* = 0.598, $\eta_{p}^{2}$ = 0.017, BF_10_ = 0.102; Alignment × Congruency × Stimulus Presentation Duration, *F*(1,59)<0.001, *p* = 0.984, $\eta_{p}^{2}$<0.001, BF_10_ = 0.203; Subject Group × Alignment × Congruency × Stimulus Presentation Duration, *F*(2,59) = 0.148, *p* = 0.862, $\eta_{p}^{2}$ = 0.005, BF_10_ = 0.186). To visualize the composite face effect across age groups, we calculated the CFE*RT* defined as [(RT_aligned congruent_ − RT_aligned incongruent_) – (RT_misaligned congruent_ − RT_misaligned incongruent_)]. The visualization data of CFE*RT* are shown in Figure 5.

## Discussion

The complete composite face paradigm was employed to investigate the development of holistic face processing in Chinese children (11–13 years), young adults (23–26 years), and older adults (56–65 years). The study results demonstrate a significant interaction between alignment and congruency, which provides evidence that faces are processed holistically when the composite face paradigm is applied to Chinese people. The results show that the holistic face processing ability of 11-year-old children has already reached an adult-like level. This result is consistent with previous studies that found holistic face processing ability to be similar from 11 years of age to adulthood in both Caucasians (Durand et al., 2007; Petrakova et al., 2018) and East Asian individuals (Sun et al., 2020) and that holistic face processing ability is similar between people aged 11 years and 60 years. Compared to the ability of holistic processing, other cognitive abilities, such as perception and memory related to faces (Crook and Larrabee, 1992; Lamont et al., 2005; Lott et al., 2005; Hildebrandt et al., 2010, 2011; Germine et al., 2011), visual working memory (Brockmole and Logie, 2013), attention (Georgiou-Karistianis et al., 2006), and general cognitive ability (e.g., inductive reasoning, episodic memory, and perceptual speed, sees Salthouse, 2009), tend to decline for these people aged 60. Combined with the findings concerning other cognitive abilities, the present results suggest that holistic faces processing is a relatively stable ability for individuals from their teens to their early sixties. Hence, the developmental trajectory of holistic face perception might differ from that of other cognitive abilities.

More importantly, the results demonstrated that older East Asian adults (56–65 years) have similar holistic face processing abilities to young East Asian adults (23–26 years). Previous findings about the stability of holistic face processing between adults and the elderly in the age range of 60–85 years were inconsistent (Boutet and Faubert, 2006; Konar et al., 2013; Wiese et al., 2013; Meinhardt-Injac et al., 2014, 2017; Cheng et al., 2016; Boutet and Meinhardt-Injac, 2018). One important reason for the inconsistency may be that many abilities, such as face recognition and attention span, decline from 60 to 85 years (Crook and Larrabee, 1992; Lamont et al., 2005; Boutet and Faubert, 2006; Hildebrandt et al., 2010; Germine et al., 2011). Therefore, the exploration of holistic face processing ability in older adults aged 60–85 years may be confounded with age-related changes in face recognition and attention-related abilities. Studies have demonstrated that the face recognition and attention-related abilities in older adults between 56–65 years are similar to those in young adults (Crook and Larrabee, 1992; Greenwood and Parasuraman, 1994). Therefore, the present study focused on a relatively narrow range of older adults (e.g., 56–65 years) to compare their holistic face processing ability with that of young adults. Indeed, for the first time, our study revealed a similar holistic face processing ability in Chinese people from about 11 years to 60 years of age, using the complete composite face paradigm.

Furthermore, in previous studies, the presentation duration of study faces was 200 ms (Konar et al., 2013) or 600 ms (Wiese et al., 2013; Meinhardt-Injac et al., 2014). Different presentation durations may affect holistic face processing (Richler et al., 2009). In this study, two presentation durations (200 ms and 600 ms) were used to make a direct comparison. The results show that the holistic face processing sensitivities were similar between the 200 ms and 600 ms presentation durations, suggesting that all age groups could complete the task well when the study stimulus was presented for both durations. Accordingly, the results indicate that it was not the different stimulus presentation durations, but the larger age range that caused the inconsistent findings about declines of holistic face processing abilities in older adults relative to younger adults in previous studies. Hence, the present findings indicate the stability of holistic face processing from children to young elderly even when processing time is limited by short presentation durations, a condition that may be expected to cause performance deficits in both young and old age groups relative to young adults.

This study also showed that – independent of alignment – the overall sensitivity for the recognition of same versus different face halves declined for older adults in comparison to younger adults, but was similar to that of children. Multiple studies have revealed that declines in general cognitive abilities, such as visual perception (Rizzo et al., 1986; Lott et al., 2005) and memory (Memon and Bartlett, 2002), all add to the decline in recognition performance, from younger to older adults. Research has also revealed that face recognition ability appears to gradually improve during childhood until just after the age of 30 (Germine et al., 2011; Meinhardt-Injac et al., 2017). Combining the results of our study with those of previous studies (Hildebrandt et al., 2010, 2011), we infer that the development of face recognition ability may increase from childhood to adulthood and then decrease from young adulthood to older adulthood and that face recognition ability may be related to changes in general cognitive ability. Certainly, this statement heeds extreme caution. In this study, the stimuli were adult faces, which did not include faces of children or older adults. Research demonstrated an own-age bias effect; that is, compared with other-age faces, people usually have an advantage in recognizing and remembering own-age faces (Bartlett and Leslie, 1986; Rhodes and Anastasi, 2012). This tendency is because it is more efficient to process an own-age face relative to other ages (Wiese et al., 2013). In this study, the inclusion of young adult faces may have been helpful for young adults to process the faces. To exclude the own-age bias effect, further studies may verify the issues by using age-matched face stimuli.

Moreover, our study also found that the holistic face processing ability of a 60-year-old East Asian is similar to that of a young East Asian adult aged around 20. Cheng et al. (2016) established that the holistic processing ability of East Asian elderly aged around 60 had not begun to decline. Combining the evidence from Cheng et al. (2016) and our results, it can be demonstrated that the holistic face processing ability of 60-year-old East Asian people is similar to that of younger adults. It should also be noted that previous studies demonstrated that the holistic face processing ability of East Asian adults is better than that of Western adults (Lewis et al., 2008; Miyamoto et al., 2011). However, we cannot infer directly whether the holistic face processing ability of 60-year-old Western individuals is similar to that of young Western adults. Further research could recruit older adults within a narrow age range (e.g., 56–65 years) to investigate whether the holistic face processing ability of Western older adults is less than that of Western adults.

In conclusion, the present study shows that when targeting a focused age range of younger elderly (mid-fifties to mid-sixties) with the composite task, no decline of holistic face processing was found although an age-related decline was present in general face recognition performance. A very similar picture is seen in children with less efficient face recognition performance relative to young adults but already mature holistic processing. Hence, holistic processing turns out to be a relatively stable ability in this age range as compared to face recognition, which was inferior in both the young and old age groups of this study relative to young adults.

## Data Availability Statement

The raw data supporting the conclusions of this article will be made available by the authors, without undue reservation.

## Ethics Statement

The studies involving human participants were reviewed and approved by The ethical committee of Zhejiang Normal University. The participants provided their written or verbal informed consent to participate in this study. Written informed consent was obtained from the individual(s) for the publication of any potentially identifiable images or data included in this article.

## Author Contributions

QY and XC: designed the experiments. GG: executed the project. LZ, QY, and XC: performed the data analysis and wrote the manuscript. LZ, QY, CC, WS, and XC: revised the manuscript. All authors reviewed the manuscript.

## Funding

This study was supported by the National Social Science Foundation of China (Grant no. 21FYYB051).

## Conflict of Interest

The authors declare that the research was conducted in the absence of any commercial or financial relationships that could be construed as a potential conflict of interest.

## Publisher’s Note

All claims expressed in this article are solely those of the authors and do not necessarily represent those of their affiliated organizations, or those of the publisher, the editors and the reviewers. Any product that may be evaluated in this article, or claim that may be made by its manufacturer, is not guaranteed or endorsed by the publisher.

## Footnotes

^1^ https://jasp-stats.org
