# Supplementary material for: The Composite Face Effect Between Young and Older Chinese Adults Remains Stable
Source: Front Psychol. 2021 Dec 9;12:743056. doi: 10.3389/fpsyg.2021.743056 (PMC8697428; doi:10.3389/fpsyg.2021.743056)
Supplement: Supplementary file 1 [file Data_Sheet_1.docx]

**Supplementary materials**

**1.** **Further analysis by age group**

**1.1 Sensitivity (A’) analysis by age group**

Despite that the four-way interaction was not significant in terms of A’, in order to compare the composite effects (CE) across age groups and across Stimulus Presentation Duration conditions, we calculated the CE as an interaction between Alignment and Congruency conditions in each group. We used three-way ANOVA for data analysis: 2 (Alignment: aligned vs. misaligned) × 2 (Congruency: congruent vs. incongruent) × 2 (Stimulus Presentation Duration: 200 ms vs. 600 ms).

As for children, results on A’ revealed that the main effect of Alignment was not significant, *F*(1,18) = 3.212, *p* = 0.090, *η_p_*^2^ = 0.151, nor was that of Stimulus Presentation Duration, *F*(1,18) = 0.297, *p* = 0.593, *η_p_*^2^ = 0.016. The main effect of Congruency was significant, *F*(1,18) = 8.376, *p* = 0.010, *η_p_*^2^ = 0.318. Importantly, the interaction between Alignment and Congruency was significant, *F*(1,18) = 5.886, *p* = 0.026, *η_p_*^2^ = 0.246. No other two-way interactions were significant (Alignment × Stimulus Presentation Duration, *F*(1,18) = 0.098, *p* = 0.757, *η_p_*^2^ = 0.005; Congruency × Stimulus Presentation Duration, *F*(1,18) = 0.013, *p* = 0.911, *η_p_*^2^ = 0.001). The three-way interaction of Alignment, Congruency, and Stimulus Presentation Duration was not significant, *F*(1,19) = 0.153, *p* = 0.701, *η_p_*^2^ = 0.008.

As for young adults, results showed that the main effect of Alignment was not significant, *F*(1,19) = 0.112, *p* = 0.742, *η_p_*^2^ = 0.006, nor was that of Stimulus Presentation Duration, *F*(1,19) = 2.615, *p* = 0.122, *η_p_*^2^ = 0.121 The main effect of Congruency was significant, *F*(1,19) = 15.485, *p* = 0.001, *η_p_*^2^ = 0.449. The interaction between Alignment and Congruency was significant, *F*(1,19) = 19.388, *p* < 0.001, *η_p_*^2^ = 0.505. No other two-way interactions were significant (Alignment × Stimulus Presentation Duration, *F*(1,19) = 1.534, *p* = 0.231, *η_p_*^2^ = 0.075; Congruency × Stimulus Presentation Duration, *F*(1,19) = 0.433, *p* = 0.518, *η_p_*^2^ = 0.022). The three-way interaction was not significant, *F*(1,19) = 0.170, *p* = 0.685, *η_p_*^2^ = 0.009.

As for older adults, only the main effect of Congruency was significant, *F*(1,22) = 6.147, *p* = 0.021, *η_p_*^2^ = 0.218. The main effects of Alignment(*F*(1,22) = 3.029, *p* = 0.096, *η_p_*^2^ = 0.121) and Stimulus Presentation Duration (*F*(1,22) = 0.158, *p* = 0.695, *η_p_*^2^ = 0.007) were not significant. The interaction between Alignment and Congruency was significant, *F*(1,22) = 16.265, *p* = 0.001, *η_p_*^2^ = 0.425. No other two-way interactions were significant (Alignment × Stimulus Presentation Duration, *F*(1,22) = 0.552, *p* = 0.465, *η_p_*^2^ = 0.024; Congruency × Stimulus Presentation Duration, *F*(1,22) = 0.046, *p* = 0.832, *η_p_*^2^ = 0.002).The three-way interaction of Alignment, Congruency, and Stimulus Presentation Duration was not significant, *F*(1,22) = 1.714, *p* = 0.204, *η_p_*^2^ = 0.072.

**1.2 Response time analysis by age group**

Despite that the four-way interaction was not significant in terms of response time, in order to further understand the holistic processing in each age group, we conducted a three-way repeated ANOVA: 2 (Alignment: aligned vs. misaligned) × 2 (Congruency: congruent vs. incongruent) × 2 (Stimulus Presentation Duration: 200 ms vs. 600 ms) for each age group.

As for children, results on response time suggested that there was no main effects of Alignment (*F*(1,18) = 4.273, *p* = 0.053, *η_p_*^2^ = 0.192) nor Stimulus Presentation Duration (*F*(1,18) = 4.273, *p* = 0.091, *η_p_*^2^ = 0.189). The main effect of Congruency was significant, (*F*(1,18) = 5.352, *p* = 0.033, *η_p_*^2^ = 0.229). There were no two-way interactions (Alignment × Congruency, *F*(1,18) < 0.001, *p* = 0.988, *η_p_*^2^ < 0.001; Alignment × Stimulus Presentation Duration, *F*(1,18) = 0.091, *p* = 0.766, *η_p_*^2^ = 0.005; Congruency × Stimulus Presentation Duration, *F*(1,18) = 1.064, *p* = 0.316, *η_p_*^2^ = 0.056). There was not a three-way interaction, *F*(1,18) = 0.165, *p* = 0.690, *η_p_*^2^ = 0.009.

As for young adults, the main effects of Alignment (*F*(1,19) = 33.601, *p* < 0.001, *η_p_*^2^ = 0.639) and Stimulus Presentation Duration (*F*(1,19) = 8.503, *p* = 0.009, *η_p_*^2^ = 0.309) were significant. The main effect of Congruency was not significant, *F*(1,19) = 1.784, *p* = 0.197, *η_p_*^2^ = 0.086. Importantly, the interaction between Alignment and Congruency was significant, *F*(1,19) = 15.981, *p* = 0.001, *η_p_*^2^ = 0.457. The other two-way interactions were not significant (Alignment × Stimulus Presentation Duration, *F*(1,19) = 0.002, *p* = 0.966, *η_p_*^2^ < 0.001; Congruency × Stimulus Presentation Duration, *F*(1,19) = 0.640, *p* = 0.433, *η_p_*^2^ = 0.033). The three-way interaction was also not significant, *F*(1,19) = 0.622, *p* = 0.440, *η_p_*^2^ = 0.032.

As for older adults, all main effects and interactions were not significant (main effect of Alignment, *F*(1,22) = 1.682, *p* = 0.208, *η_p_*^2^ = 0.071; main effect of Congruency, *F*(1,22) = 0.068, *p* = 0.797, *η_p_*^2^ = 0.003; main effect of Stimulus Presentation Duration, *F*(1,22) = 0.008, *p* = 0.929, *η_p_*^2^ < 0.001; Alignment × Congruency, *F*(1,22) = 0.031, *p* = 0.863, *η_p_*^2^ = 0.001; Alignment × Stimulus Presentation Duration, *F*(1,22) = 0.011, *p* = 0.916, *η_p_*^2^ = 0.001; Congruency × Stimulus Presentation Duration, *F*(1,22) = 0.509, *p* = 0.483, *η_p_*^2^ = 0.023; Alignment × Congruency × Stimulus Presentation Duration, *F*(1,22) = 0.001, *p* = 0.970, *η_p_*^2^ < 0.001).
